# Supplementary material for: Clinical study outcomes in IgA nephropathy: A systematic literature review and narrative synthesis
Source: PLoS One. 2025 Jun 10;20(6):e0323530. doi: 10.1371/journal.pone.0323530 (PMC12151485; doi:10.1371/journal.pone.0323530)
Supplement: S1 Table — (DOCX) [file pone.0323530.s001.docx]

**Supplementary Table S1**: Ovid Embase search string

|  | Search terms | October 18 2021 | December 11 2023 |
| --- | --- | --- | --- |
| 1 | immunoglobulin a nephropathy/ | 13,118 | 16,271 |
| 2 | Glomerulonephritis/ and Immunoglobulin A/ | 1,422 | 1,729 |
| 3 | (iga adj3 nephropath$).ti,ab,kw | 10,264 | 12,514 |
| 4 | iga glomerulonephritis.ti,ab,kw. | 432 | 461 |
| 5 | igan.ti,ab,kw. | 4353 | 5,782 |
| 6 | (immunoglobulin a adj3 (nephropath$ or glomerulonephritis)).ti,ab,kw. | 1,533 | 2,057 |
| 7 | immunoglobulin iga nephropathy.ti,ab,kw. | 7 | 9 |
| 8 | or/1-7 | 15,308 | 18,824 |
| 9 | Randomized controlled trial/ | 679,938 | 797,291 |
| 10 | Controlled clinical trial/ | 464,183 | 471,699 |
| 11 | random$.ti,ab. | 1,715,210 | 2,007,768 |
| 12 | randomization/ | 91,975 | 98,893 |
| 13 | intermethod comparison/ | 276,076 | 303,079 |
| 14 | placebo.ti,ab. | 330,812 | 369,668 |
| 15 | (compare or compared or comparison).ti. | 548,446 | 612,066 |
| 16 | ((evaluated or evaluate or evaluating or assessed or assess) and (compare or compared or comparing or comparison)).ab. | 2,382,477 | 2,828,897 |
| 17 | (open adj label).ti,ab. | 91,651 | 111,588 |
| 18 | ((double or single or doubly or singly) adj (blind or blinded or blindly)).ti,ab. | 249,347 | 276,970 |
| 19 | double blind procedure/ | 188,718 | 213,603 |
| 20 | parallel group$1.ti,ab. | 28,237 | 32,632 |
| 21 | (crossover or cross over).ti,ab. | 113,052 | 125,907 |
| 22 | ((assign$ or match or matched or allocation) adj5 (alternate or group$1 or intervention$1 or patient$1 or subject$1 or participant$1)).ti,ab. | 364,782 | 421,551 |
| 23 | (assigned or allocated).ti,ab. | 429,887 | 498,200 |
| 24 | (controlled adj7 (study or design or trial)).ti,ab. | 390,200 | 457,888 |
| 25 | (volunteer or volunteers).ti,ab. | 261,420 | 284,913 |
| 26 | human experiment/ | 556,517 | 651,308 |
| 27 | trial.ti. | 341,049 | 409,468 |
| 28 | or/9-27 | 5,544,723 | 6,422,749 |
| 29 | (random$ adj sampl$ adj7 (cross section$ or questionnaire$1 or survey$ or database$1)).ti,ab. not (comparative study/ or controlled study/ or randomi?ed controlled.ti,ab. or randomly assigned.ti,ab.) | 8,736 | 9,692 |
| 30 | Cross-sectional study/ not (randomized controlled trial/ or controlled clinical study/ or controlled study/ or randomi?ed controlled.ti,ab. or control group$1.ti,ab.) | 285,457 | 370,543 |
| 31 | (((case adj control$) and random$) not randomi?ed controlled).ti,ab. | 19,032 | 21,855 |
| 32 | (Systematic review not (trial or study)).ti. | 188,755 | 268,401 |
| 33 | (nonrandom$ not random$).ti,ab. | 17,310 | 19,138 |
| 34 | Random field$.ti,ab. | 2,595 | 2,995 |
| 35 | (random cluster adj3 sampl$).ti,ab. | 1,387 | 1,605 |
| 36 | (review.ab. and review.pt.) not trial.ti. | 931,563 | 1,152,894 |
| 37 | we searched.ab. and (review.ti. or review.pt.) | 38,644 | 50,613 |
| 38 | update review.ab. | 118 | 137 |
| 39 | (databases adj4 searched).ab. | 45,856 | 64,421 |
| 40 | (rat or rats or mouse or mice or swine or porcine or murine or sheep or lambs or pigs or piglets or rabbit or rabbits or cat or cats or dog or dogs or cattle or bovine or monkey or monkeys or trout or marmoset$1).ti. and animal experiment/ | 1,124,811 | 1,232,922 |
| 41 | Animal experiment/ not (human experiment/ or human/) | 2,360,518 | 2,590,017 |
| 42 | or/29-41 | 3,805,263 | 4,408,946 |
| 43 | 28 not 42 | 4,919,706 | 5,663,656 |
| 44 | exp clinical trial/ | 1,638,185 | 1,864,096 |
| 45 | (clinical adj2 trial$).ti,ab,kw. | 602,380 | 722,950 |
| 46 | exp "clinical trial (topic)"/ | 370,699 | 451,053 |
| 47 | (phase i$5 or phase ii$5 or phase iii$5 or phase iv$5 or phase 1$5 or phase 2$5 or phase 3$5 or phase 4$5).ti,ab,kw. | 391,656 | 457,599 |
| 48 | (phase l$5 or phase ll$5 or phase lll$5 or phase lv$5).ti,ab,kw. | 22,260 | 24,650 |
| 49 | or/44-48 | 2,406,177 | 2,782,602 |
| 50 | (((systematic or state-of-the-art or scoping or literature or umbrella) adj (review* or overview* or assessment*)) or "review* of reviews" or meta-analy* or metaanaly* or ((systematic or evidence) adj1 assess*) or "research evidence" or metasynthe* or meta-synthe*).tw. or systematic review/ or "systematic review (topic)"/ or meta analysis/ or "meta analysis (topic)"/ | 705,125 | 919,363 |
| 51 | limit 50 to yr="2016 -Current" | 383,292 | 594,601 |
| 52 | 8 and (43 or 49 or 51) | 2,809 | 3,842 |
| 53 | limit 52 to (english language and yr="1980 -Current") | 2,639 | 3,650 |
| 54 | case report/ | 2,666,484 | 2,946,623 |
| 55 | case report.ti. | 328,286 | 391,529 |
| 56 | letter/ or editorial/ | 1,804,871 | 1,975,736 |
| 57 | 53 not (54 or 55 or 56 or 40 or 41) | **2,444** | 3,349 |
| 58 | limit 57 to dc="20211018-20230327" | - | **904** |
|  | **Total** | **3,348** | |
