# Supplementary material for: Clinical study outcomes in IgA nephropathy: A systematic literature review and narrative synthesis
Source: PLoS One. 2025 Jun 10;20(6):e0323530. doi: 10.1371/journal.pone.0323530 (PMC12151485; doi:10.1371/journal.pone.0323530)
Supplement: S2 Table — (DOCX) [file pone.0323530.s002.docx]

**Supplementary Table S2:** PubMed search string

|  | Search terms | October 18 2021 | December 11 2023 |
| --- | --- | --- | --- |
| 1 | ((((((Glomerulonephritis, IGA[mesh:noexp]) OR (Glomerulonephritis[mesh:noexp] AND Immunoglobulin A[mesh:noexp]) OR (iga[tiab] AND nephropath*[tiab]) OR (iga [ot] AND nephropath*[ot]) OR ("iga glomerulonephritis"[tiab]) OR ("iga glomerulonephritis"[ot]) OR (igan[tiab] OR igan[ot]) OR ("immunoglobulin a"[tiab] AND (nephropath*[tiab] OR glomerulonephritis[tiab])) OR ("immunoglobulin a"[ot] AND (nephropath*[ot] OR glomerulonephritis[ot]))) AND (("randomized controlled trial"[Publication Type] OR "controlled clinical trial"[Publication Type] OR "randomized"[Title/Abstract] OR "placebo"[Title/Abstract] OR "drug therapy"[MeSH Subheading] OR "randomly"[Title/Abstract] OR "trial"[Title/Abstract] OR "groups"[Title/Abstract]) OR ("Clinical Trial"[Publication Type:noexp] OR "clinical trial, phase i"[Publication Type] OR "clinical trial, phase ii"[Publication Type] OR "clinical trial, phase iii"[Publication Type] OR "clinical trial, phase iv"[Publication Type] OR "controlled clinical trial"[Publication Type] OR "multicenter study"[Publication Type] OR "randomized controlled trial"[Publication Type] OR "Clinical Trials as Topic"[MeSH Terms:noexp] OR "clinical trials, phase i as topic"[MeSH Terms:noexp] OR "clinical trials, phase ii as topic"[MeSH Terms:noexp] OR "clinical trials, phase iii as topic"[MeSH Terms:noexp] OR "clinical trials, phase iv as topic"[MeSH Terms:noexp] OR "controlled clinical trials as topic"[MeSH Terms:noexp] OR "randomized controlled trials as topic"[MeSH Terms:noexp] OR "early termination of clinical trials"[MeSH Terms:noexp] OR "multicenter studies as topic"[MeSH Terms:noexp] OR "Double-Blind Method"[MeSH Terms] OR (("randomised"[Title/Abstract] OR "randomized"[Title/Abstract]) AND ("trial"[Title/Abstract] OR "trials"[Title/Abstract])) OR (("single"[Title/Abstract] OR "double"[Title/Abstract] OR "doubled"[Title/Abstract] OR "triple"[Title/Abstract] OR "tripled"[Title/Abstract] OR "treble"[Title/Abstract] OR "treble"[Title/Abstract]) AND ("blind*"[Title/Abstract] OR "mask*"[Title/Abstract])) OR ("4 arm"[Title/Abstract] OR "four arm"[Title/Abstract])) OR (((("review"[Title/Abstract] OR "review"[Publication Type] OR "meta-analysis"[Title/Abstract] OR "meta-analysis"[Publication Type]) NOT ("letter"[Publication Type] OR "comment"[Publication Type] OR "editorial"[Publication Type])) OR ("systematic review"[Publication Type])) AND (("2016"[Date - Publication] : "2022"[Date - Publication]))))) AND english[language]) NOT ((case reports[pt] OR case report[ti]) OR (animals[mesh] NOT humans[mesh]))) NOT (letter[pt] OR comment[pt] OR editorial[pt])) | **2,116** | **403** |
|  | **Total** | **2,519** | |
