# Supplementary material for: Clinical study outcomes in IgA nephropathy: A systematic literature review and narrative synthesis
Source: PLoS One. 2025 Jun 10;20(6):e0323530. doi: 10.1371/journal.pone.0323530 (PMC12151485; doi:10.1371/journal.pone.0323530)
Supplement: S3 Table — (DOCX) [file pone.0323530.s003.docx]

**Supplementary Table S3**: Cochrane library CENTRAL database and database of Systematic Reviews search strings

|  | Search terms | October 18 2021 | December 11 2023 |
| --- | --- | --- | --- |
| Cochrane Library CENTRAL database Search String | | |  |
| 1 | MeSH descriptor: [Glomerulonephritis, IGA] explode all trees | 252 | 316 |
| 2 | MeSH descriptor: [Immunoglobulin A] this term only | 268 | 350 |
| 3 | MeSH descriptor: [Immunoglobulin A] this term only | 775 | 923 |
| 4 | (iga NEAR/3 nephropath*):ti,ab,kw | 585 | 736 |
| 5 | ("IgA glomerulonephritis"):ti,ab,kw | 15 | 15 |
| 6 | ("IgAN"):ti,ab,kw | 253 | 379 |
| 7 | ("immunoglobulin a" AND (nephropath* or glomerulonephritis)):ti,ab,kw | 288 | 413 |
| 8 | #1 OR (#2 AND #3) OR #4 OR #5 OR #6 OR #7 with Publication Year from 1980 to 2021, in Trials | **677** | - |
| 9 | #1 or (#2 AND #3) or #4 or #5 or #6 or #7 with Publication Year from 2021 to 2023, in Trials | - | **176** |
|  | **Total** | **853** | |
| **Cochrane Library Database of Synthetics Reviews Search string** | | |  |
| 1 | MeSH descriptor: [Glomerulonephritis, IGA] explode all trees | 252 | 462 |
| 2 | MeSH descriptor: [Glomerulonephritis] this term only | 268 | 1,738 |
| 3 | MeSH descriptor: [Immunoglobulin A] this term only | 775 | 14,415 |
| 4 | (iga NEAR/3 nephropath*):ti,ab,kw | 585 | 751 |
| 5 | ("IgA glomerulonephritis"):ti,ab,kw | 15 | 15 |
| 6 | ("IgAN"):ti,ab,kw | 253 | 394 |
| 7 | ("immunoglobulin a" AND (nephropath* or glomerulonephritis)):ti,ab,kw | 288 | 420 |
| 8 | 1 OR (#2 AND #3) OR #4 OR #5 OR #6 OR #7 with Cochrane Library publication date Between Jan 2016 and Oct 2021, in Cochrane Reviews | **1** | - |
| 9 | #1 or (#2 AND #3) or #4 or #5 or #6 or #7 with Cochrane Library publication date Between Nov 2021 and Dec 2023, in Cochrane Reviews | - | **2** |
|  | **Total** | **3** | |
