# Supplementary material for: Clinical study outcomes in IgA nephropathy: A systematic literature review and narrative synthesis
Source: PLoS One. 2025 Jun 10;20(6):e0323530. doi: 10.1371/journal.pone.0323530 (PMC12151485; doi:10.1371/journal.pone.0323530)
Supplement: S4 Table — (DOCX) [file pone.0323530.s004.docx]

**Supplementary Table S4:** Study design and population characteristics of selected studies

| **Author (year); Trial ID** | **Study design** | **Country** | **Treatment (N)** | **Study population/ inclusion criteria** |
| --- | --- | --- | --- | --- |
| Shima et al.(1); JSKDC01/ C000000006 | Prospective  Open-label  RCT | Japan | Lisinopril [0.1 mg/kg] (N= 31) | - Aged 2–18 years - Biopsy-proven IgAN - Early morning UPCR >0.2 g/g |
|  |  |  | Lisinopril [0.1 mg/kg] + losartan [0.7 mg/kg] (N=31) |  |
| Kohagura et al.(2); ACTRN12610000516088 | Phase 2/ 3  Open-label  RCT | Japan | Standard therapy (steroid pulse, prednisolone, and tonsillectomy) (N=37) | - Aged 15-70 years - Histologically proven IgAN - PCR ≥0.5 g/gCr |
|  |  |  | Standard therapy (steroid pulse, prednisolone, and tonsillectomy) + candesartan [2-8 mg/day] (N=40) |  |
| Jo et al.(3) | Prospective  Open-label  RCT | South Korea | Valsartan [40 mg] (N=23) | - Aged >18 years - Biopsy-proven IgAN - Persistent proteinuria defined as a UP/C of 0.3 to 1.0 g/g for 2 months |
|  |  |  | Valsartan [80 mg] (N=20) |  |
| Li et al.(4); NCT01225445 | Phase 3  Open-label  RCT | China | Ramipril (N=30) | - Aged 18-65 years - Biopsy-confirmed IgAN - Proteinuria <0.5 g per day |
|  |  |  | No treatment (N=30) |  |
| Woo et al.(5) | Multicenter  Open-label  RCT | Singapore | Losartan [200 mg/day] (N=63) | - Aged >18 years - PCR >1 g/g or more and or CKD Stage 3 |
|  |  |  | Losartan [100 mg/day] (N=43) |  |
|  |  |  | Enalapril [20 mg/day] (N=61) |  |
|  |  |  | Enalapril [10 mg/day] (N=40) |  |
| Shimizu et al.(6) | RCT  Parallel-group  Open-label | Japan | Losartan [12.5 mg/day] (N=18) | - Aged >18 years - Biopsy-proven IgAN - Normotensive - Persistent proteinuria ≥0.4 g/day despite treatment with antiplatelet agents for >3 months without ACEis or ARBs |
|  |  |  | Antiplatelet (N=18) |  |
| Coppo et al.(7); IgACE | Placebo-controlled  Randomized  Multicenter  Double-blind | Europe | Benazepril [0.2 mg/kg] (N=32) | - Aged 3-35 years - Biopsy-proven IgAN - Proteinuria 1-3.4 g/day per 1.73 m² |
|  |  |  | Placebo (N=34) |  |
| Horita et al.(8) | Prospective  Single-center  RCT  Open-label | Japan | Prednisolone [5-30 mg/dL] + losartan [50 mg/day] (N=22) | - Aged >18 years - Patients with normal BP - Persistent mild-to-moderate proteinuria of 1.0–2.6 g/day |
|  |  |  | Prednisolone [5-30 mg/dL] (N=18) |  |
| Woo et al.(9) | RCT  Open-label | Singapore | Enalapril [5-10 mg/day] and/or losartan [50-100 mg/day] (N=37) | - Aged >18 years - Biopsy-proven IgAN - Proteinuria ≥1g/day and/or renal impairment defined as SCr >1.6mg/dL |
|  |  |  | Control (N=38) |  |
| Li et al.(10); HKVIN | Double-blind  RCT | Hong Kong | Valsartan [80 mg/day] (N=54) | - Aged >18 years - Proteinuria >1 g/day - Primary IgAN - Recent treatment with ARBs or ACEis (within 4 weeks) |
|  |  |  | Placebo (N=55) |  |
| Horita et al.(11) | Prospective  RCT  Parallel-group  Open-label | Japan | Temocapril [1 mg/day] (N=14) | - Aged >18 years - Biopsy-proven IgAN - Normotensive - Persistent mild-to-moderate proteinuria (≥0.4-1.6 g/day) - On antiplatelet agents for 3 months before without ACEi or ARB |
|  |  |  | Losartan [12.5 mg/day] (N=16) |  |
|  |  |  | Temocapril [1 mg/day] + losartan [12.5 mg/day] (N=13) |  |
| Horita et al.(12) |  |  | Temocapril [1 mg/day] (N=10) |  |
|  |  |  | Losartan [12.5 mg/day] (N=10) |  |
|  |  |  | Temocapril [1 mg/day] + losartan [12.5 mg/day] (N=11) |  |
| Kanno et al.(13) | Prospective  Open-label  RCT | Japan | Temocapril [1-2 mg/day] or trandolapril [mg/day] (N=26) | - Aged >18 years - Biopsy-proven IgAN |
|  |  |  | Amlodipine [2.5-5 mg/day] (N=23) |  |
| Kim et al.(14) | Double-blind  RCT  Crossover trial | South Korea | Ramipril [5 mg/day] (N=19, IgAN only) | - Biopsy-proven IgAN or type 2 diabetic nephropathy - BP <130/80 mmHg - Receiving more than 5 mg ramipril once daily for at least 6 months - 24h-PER <1.0 g/day - No previous history of steroid or cytotoxic treatment within the last 6 months |
|  |  |  | Ramipril [5 mg/day] + candesartan [4 mg/day] (N=19, IgAN only) |  |
|  |  |  | Placebo (N=19, IgAN only) |  |
| Praga et al.(15) | RCT  Prospective  Single-center  Open-label | Spain | Enalapril [5-40 mg/day] (N=23) | - Biopsy-proven IgAN - 24h-PER ≥0.5 g/d |
|  |  |  | Control (N=21) |  |
| Nakamura et al.(16) | Parallel  Controlled  RCT  Open-label | Japan | Verapamil [120 mg/day] (N=8) | - Aged 18-54 years - Biopsy-proven IgAN - BP <140/90 mmHg - Proteinuria <3g/day - No previous immunosuppressive, antihypertensive or anti-inflammatory drugs |
|  |  |  | Trandolapril [2 mg/day] (N=8) |  |
|  |  |  | Candesartan [8 mg/day] (N=8) |  |
|  |  |  | Placebo (N=8) |  |
| Woo et al.(17) | Prospective  Parallel  RCT  Controlled  Open-label | Singapore | Enalapril [5 mg/day] and/or losartan [50 mg/day] (N=21) | - Biopsy-proven primary IgAN - Proteinuria ≥1 g - Well controlled BP - No previous treatment with aspirin, warfarin, dipyridamole, steroids, cytotoxics, immunosuppressants, CCB |
|  |  |  | Control (N=20) |  |
|  |  |  | Enalapril [5 mg/day] and/or losartan [50 mg/day] -responders (N=10) |  |
|  |  |  | Enalapril [5 mg/day] and/or losartan [50 mg/day] -non-responders (N=11) |  |
| Maschio et al.(18) | Double-blind  Crossover  RCT  Placebo-controlled | Italy | Fosinopril [20 mg/day] (N=39) | - Aged 18-54 years - Biopsy-proven IgAN - Proteinuria ≥1 g/24h - BP <140/90mmHg |
|  |  |  | Placebo (N=39) |  |
| Park et al.(19) | Prospective  RCT  Open-label | South Korea | Control (N=22) | - Aged ≥18 years - Hypertensive IgAN patients - 24h-PER >1 g/day - No previous treatment with oral corticosteroids, and concomitant use of agents that may affect BP and proteinuria except a- or b-blockers, diuretics, and nitrates |
|  |  |  | Losartan [50 mg/day] (N=20) |  |
|  |  |  | Amlodipine [5 mg/day] (N=16) |  |
| Barratt et al.(20); NCT04564339 | RCT  Open-label | International | Ravulizumab (N=43) | - Aged 18–75 years - Biopsy-confirmed IgAN - Proteinuria ≥1g/d - On stable maximally tolerated RASi with stable blood pressure ≥3 months |
|  |  |  | Placebo (N=23) |  |
| Zhang et al.(21); NCT03373461 | RCT  Double-blind  Parallel | International | Iptacopan [10 mg] (N=20) | - Female and male patients ≥18 years - Biopsy-verified IgAN - Measured or eGFR ≥30 mL/min per 1.73 m² - Urine protein ≥1 g/24hr at screening and ≥0.75 g/24h after the run- in period - All patients must have been on supportive care including a maximally tolerated dose of ACEi or ARB therapy for the individual, antihypertensive therapy or diuretics for at least 90 days before dosing |
|  |  |  | Iptacopan [50 mg] (N=19) |  |
|  |  |  | Iptacopan [100 mg] (N=22) |  |
| Barratt et al.(22)**;** NCT03373461 |  |  | Iptacopan [200 mg] (N=26) |  |
|  |  |  | Placebo (N=25) |  |
| NCT03373461 trial record(23)**;** NCT03373461 |  |  |  |  |
| Lafayette et al.(24); NefIgArd/ NCT03643965 | Phase 3  Double-blind  RCT | International | TRF-budesonide [16 mg/day] (N=97) | - >18 years of age - Biopsy-confirmed primary IgAN - Persistent proteinuria (UPCR ≥0.8 g/g or proteinuria ≥1 g/24h) despite optimized supportive care - eGFR of ≥35 to ≤90 ml/min per 1.73 m² |
| Barratt et al. (25); NefIgArd/ NCT03643965 |  |  |  |  |
|  |  |  | Placebo (N=102) |  |
| Barratt et al.(26); NefIgArd/ NCT03643965 |  |  |  |  |
| NCT03643965 trial record(27) |  |  |  |  |
| Han et al. (28); NCT02981212 | Phase 4  Open-label  RCT | South Korea | MMF + prednisolone (N=24) | - 19 to 65 years of age - Biopsy-proven IgAN - UPCR >0.75 g/day - eGFR between 20 and 50 mL/min/1.73 m² |
|  |  |  | Supportive Care (RAS blockers) (N=20) |  |
| Jung et al. (29); NCT02981212 |  |  | MMF + corticosteroid (N=48 both arms) |  |
|  |  |  | Placebo (N=48 both arms) |  |
| Liang et al.(30); NCT02160132 | Phase 2  Prospective  RCT  Non-blind study | China | 1–23 group (N=34)  methylprednisolone + prednisone | - 14–65 years - Clinical evaluation and renal biopsy diagnostic for primary IgAN, presenting with crescents - Mean urinary protein excretion of 0.5–3.5 g/24h - eGFR ≥50 ml/min/1.73 m^2^ |
|  |  |  | 1–35 group (N=34)  methylprednisolone + prednisone |  |
| Sun et al.(31) | Prospective  Double-blind  RCT | China | Supportive care (N=71) | - 18-55 years of age - Blood pressure ≤130/90 mmHg after 3-month treatment of maximum tolerable dose of ACEI/ ARB - eGFR ≥45ml/min/1.73m² - Urinary protein ≥1.0 g/day or UACR ≥0.8 g/g |
|  |  |  | Supportive care + fluticasone (N=71) |  |
| Hou et al.(32); NCT01854814 | Prospective  Open-label  RCT | China | MMF + supportive care (losartan and erythropoietin, antihypertensive, and statin if needed) (N=85) | - 18 to 70 years of age - Biopsy-proven IgAN - Urinary protein excretion rate (UPER) greater than 1 g/d - eGFR less than 60 mL/min/1.73m^2^ or persistent hypertension (blood pressure greater than 140/90 mmHg) or need of an antihypertensive drug |
|  |  |  | Supportive care (losartan and erythropoietin, antihypertensive, and statin if needed) (N=85) |  |
| Lv et al.(33); NCT04291781 | Phase 2  Double-blind  RCT | China | Placebo (N=14) | - Biopsy-confirmed primary IgAN - Proteinuria ≥0.75 g/day - eGFR ≥35 ml/min per 1.73 m² |
| Lv et al.(34); NCT04291781 |  |  | Telitacicept [160 mg] (N=16) |  |
|  |  |  | Telitacicept [240 mg] (N=14) |  |
| Ni et al.(35); ISRCTN97636235 | Prospective  Open-label  RCT | China | Leflunomide [20-40 mg/day]+ prednisone [0.5-0.8 mg/kg/day] (N=59) | - Aged 18–65 years - Biopsy-confirmed primary IgAN |
|  |  |  | Prednisone [0.5-0.8 mg/kg/day] (N=49) |  |
| Rauen et al.(36); STOP-IgAN/ NCT00554502 | Phase 3  Prospective  Open-label  RCT with a two-group, parallel, group-sequential design | Germany | Supportive therapy (RAS blockade) (N=80) | - Aged 18-70 years - Primary IgAN confirmed on biopsy, proteinuria > 0.75 g/day - No secondary and rapidly progressive, crescentic IgAN, other chronic renal diseases - No prior immunosuppressive therapy |
|  |  |  | Supportive therapy (RAS blockade) + immunosuppression (N=82) |  |
| Rauen et al.(37); STOP-IgAN/ NCT00554502 |  |  | High-eGFRe: supportive therapy (RAS blockade) (N=54) |  |
|  |  |  | High-eGFRe: supportive therapy (RAS blockade) + immunosuppression (N=55) |  |
|  |  |  | Low eGFRf: supportive therapy (RAS blockade) (N=26) |  |
|  |  |  | Low eGFRf: supportive therapy (RAS blockade) + immunosuppression (N=27) |  |
| Lennartz et al.(38); STOP-IgAN/ NCT00554502 |  |  | Single RAS blockade (N=82) |  |
|  |  |  | Dual RAS blockade (N=30) |  |
| Liu et al.(39); NCT02942381 | Phase 2  Prospective  Double-blind  RCT | China | Hydroxychloroquine sulfate [400 mg/day] (N=30) | - Aged 18-75 years - Biopsy-proven primary IgAN - eGFR >30 mL/min/1.73 m² - Proteinuria 0.75-3.5 g/day despite receiving an MTD of RASi for at least 3 months |
|  |  |  | Placebo (N=30) |  |
| Fellström et al.(40); NEFIGAN/ NCT01738035 | Phase 2  Double-blind  RCT  Placebo-controlled trial | Multinational | Placebo (N=50) | - Aged ≥18 years - Biopsy-confirmed primary IgAN - Overt proteinuria for the run-in phase - eGFR ≥45 mL/min/1.73 m² - UP/C ≥0.5 g/g or 24h-PER ≥0.75 g/day |
|  |  |  | TRF-budesonide [8 mg/day] (N=51) |  |
|  |  |  | TRF-budesonide [16 mg/day] (N=48) |  |
| Hirai et al.(41) | Multicenter  RCT  Open-label  Parallel-design study | Japan | Standard therapy (steroid pulse and tonsillectomy) + mizoribine (N=21) | - Aged >16 years - Biopsy-confirmed IgAN - 24h-PER > 0.5 g/day |
|  |  |  | Standard therapy (steroid pulse and tonsillectomy) (N=21) |  |
| Hou et al.(42); NCT01269021 | Multicenter  Prospective  RCT  Open-label | China | MMF + prednisone (N=86) | - Aged 18-65 years - Biopsy-proven IgAN - Cellular and fibrocellular crescents involving 10% to <50% of glomeruli - 24h-PER ≥1.0 g/day |
|  |  |  | Prednisone (N=88) |  |
| Lafayette et al.(43); NCT00498368 | Phase 4  Open-label  RCT | US | Rituximab [1 g] + standard therapy (fish oil with ACEi/ARBs) (N=17) | - Aged ≥18 years - Biopsy-proven IgAN - No previous rituximab or immunosuppressive therapy including prednisone or corticosteroids |
|  |  |  | Standard therapy (fish oil with ACEi/ARBs) (N=17) |  |
| Kim et al. (44); TESTING/ NCT01560052 | Multicenter  Double-blind  RCT | China  Australia | Methylprednisolone (N=257) | - Aged ≥18 years - Biopsy-confirmed primary IgAN - eGFR between 20-120 mL/min/1.73 m² - 24h-PER >1 g/day |
| Kim et al.(45); TESTING/ NCT01560052 |  |  | Placebo (N=246) |  |
| Lv et al.(46); TESTING/ NCT01560052 |  |  | Full-dose methylprednisolone protocol (N=136) |  |
|  |  |  | Full-dose placebo protocol (N=126) |  |
|  |  |  | Reduced dose methylprednisolone protocol  (N=121) |  |
|  |  |  | Reduced dose placebo protocol (N=120) |  |
| Lv et al.(46); TESTING/ NCT01560052 |  |  |  |  |
| Min et al.(47) | Prospective  Single-center  Open-label  RCT | China | Prednisone [0.8 mg/kg/day] (N=45) | - Aged 18-65 years - 24h-PER ≥1.0 g/day - eGFR ≥30 ml/min/1.73m² |
|  |  |  | Leflunomide [20-40 mg/day] + prednisone [0.8 mg/kg/day] (N=40) |  |
| Kim et al.(48); NCT01224028 | Phase 2  Prospective  Double-blind  RCT | South Korea | Placebo (N=20) [treatment phase] | - Aged 18-70 years - Biopsy-proven IgAN - eGFR ≥45 ml/min/1.73 m² - UA/C 0.3-3.0 g/g |
|  |  |  | Tacrolimus [0.1 mg/kg/day] (N=20) [treatment phase] |  |
| Yu et al.(49); NCT01224028 |  |  | Tacrolimus [0.1 mg/kg/day] (N=20) [FU phase] |  |
|  |  |  | Placebo (N=20) [FU phase] |  |
| Masutani et al.(50); UMIN000000593 | Multicenter  Open-label  Prospective  RCT | China | MP [500 mg] + prednisolone [30 mg/day] (N=20) | - Biopsy-proven IgAN - Aged 15–59 years - Previously reported glomerular score of 5 or higher - No previous treatment with corticosteroids, MZR or other immunosuppressants |
|  |  |  | MP [500 mg] + prednisolone [30 mg/day] + mizoribine [150 mg/day] (N=20) |  |
| Hogg et al.(51); NCT00318474 | Phase 3,  Double-blind  RCT  Placebo-controlled trial | Canada  US | MMF [25-36 mg/kg/day] (N=7 at FU) | - Aged 7-70 years - Biopsy-confirmed IgAN - UP/C ≥0.6 g/g (males) or ≥0.8 g/g (females) - eGFR ≥50 mL/min/1.73 m² - No current or prior treatment with MMF or azathioprine |
|  |  |  | Placebo (N=10 at FU) |  |
| Liu et al.(52) | Prospective  Single-center  RCT  Open-label | China | MMF [0.75-1 g/day] + prednisone [0.8-1 mg/kg/day] (N=42) | - Aged 18-70 years - Pathological findings suggesting IgAN - 24h-PER ≥1.0 g/day |
|  |  |  | Cyclophosphamide [0.8-1 g/month] + prednisone [0.8-1 mg/kg/day] (N=42) |  |
| Liu et al.(53) | Prospective  Single-center  RCT  Open-label | China | MP [0.8 mg/kg/day] + CSA [3 mg/kg/day] (N=23) | - Aged 18 to 69 years - Biopsy-proven IgAN - 24h-PER >1.0 g/day - eGFR >30 mL/min/1.73 m² - No consecutive treatment for more than 3 months with corticosteroids/ immunosuppressive drugs within the previous year |
|  |  |  | MP [0.8 mg/kg/day] (N=25) |  |
| Pozzi et al.(54); NCT00755859/ NCT01392833 | Phase 3  Multicenter  Open-label  RCT | Italy  Switzerland | MP [1 g] + prednisone [0.5 mg/kg] + azathioprine [1.5 mg/kg/day] (N=101) | - Adult patients - IgAN diagnosis - 24h-PER ≥1.0 g/day for at least 3 months - No steroid or cytotoxic drug treatment during the previous 3 years |
|  |  |  | MP [1 g] + prednisone [0.5 mg/kg] (N=106) |  |
| Pozzi et al.(55); NCT00755859/ NCT01392833 |  |  | MP [1 g] + prednisone [0.5 mg/kg] + azathioprine [1.5 mg/kg/day] (N=20) |  |
|  |  |  | MP [1 g] + prednisone [0.5 mg/kg] (N=26) |  |
| Liu et al.(56) | Prospective  Double-arm  RCT  Controlled trial  Open-label | China | Prednisone [0.8 mg/kg/day] + leflunomide [20-50 mg/day] (N=20) | - Adult patients - 24h-PER ≥3.5 g/day - Serum albumin <30 g/L |
|  |  |  | Prednisone [0.8 mg/kg/day] + MMF [1 g/day] (N=20) |  |
| Tang et al.(57) & Tang et al.(58); NCT00863252 | Phase 4  Prospective  Multicenter  Open-label  RCT | China | MMF [1.5-2 g/day] (N=20) | - Adult patients - Histologically-confirmed IgAN - 24h-PER >1 g/day |
|  |  |  | Conventional therapy (ACEi/ARB) (N=20) |  |
| Lv et al.(59); NCT00378443 | Prospective  Open-label  Single-center  RCT | China | Prednisone [0.8-1 mg/kg/day] + cilazapril [2.5-5 mg/day] (N=33) | - Aged of 18-65 years - Biopsy-proven IgAN - 24h-PER 1-5 g/day - eGFR >30 mL/min/1.73 m² - No treatment with steroids or cytotoxic drugs during the previous 1 year |
|  |  |  | Cilazapril [2.5-5 mg/day] (N=30) |  |
| Manno et al.(60) | Prospective  Open-label  RCT | Italy | Ramipril [2.5 mg/day] (N=49) | - Biopsy-confirmed IgAN - Histological grade G2 (moderate) lesions - Aged 16-70 years - 24h-PER ≥1.0 g/day - eGFR ≥50 ml/min/1.73 m² - No treatment with corticosteroids or immunosuppressive drugs in the previous 2 years |
|  |  |  | Prednisone [0.2 mg/kg/day] + ramipril [2.5 mg/day] (N=48) |  |
| Koike et al.(61) | Single-center  Open-label  RCT  Controlled trial | Japan | Prednisolone [20-30 mg/day] (N=24) | - Adult patients - IgAN diagnosis |
|  |  |  | Dipyridamole [150 mg/day] or zilazep [300 mg/day] (N=24) |  |
| Lou et al.(62) | Prospective  Single-center  RCT  Open-label | China | Leflunomide [20 mg/day] (N=24) | - Biopsy-proven IgAN - Aged 18–65 years - 24h-PER 1.0-3.0 g/day - No use of other immunosuppressive agents |
|  |  |  | Control (N=22) |  |
| Frisch et al.(63) | Multicenter  Double-blind  RCT  Placebo-controlled | US | MMF (N=17) | - Aged 18–75 years - 24h-PER ≥1 g/day - Presence of glomerulosclerosis, tubulointerstitial fibrosis and/or crescent formation in ≥25% of the biopsy sample - No corticosteroids, or other immunosuppressive agents <6 months prior to randomization |
|  |  |  | Placebo (N=15) |  |
| Maes et al.(64) | Prospective  Single-center  RCT  Placebo-controlled  Double-blind | Belgium | MMF [1000 mg bid] (N=21) | - Aged 18 years - Biopsy-proven IgAN - 24h-PER >1 g/day - No other immunosuppressive drugs or any study drug during the last 6 months |
|  |  |  | Placebo (N=13) |  |
| Pozzi et al.(65) & Pozzi et al.(66) | Prospective  RCT  Multicenter  Open-label | Italy | MP [1 g] + prednisone [0.5 mg/kg] + supportive therapy (diuretics, antihypertensive and antiplatelet agents) (N=43) | - Aged 15–69 years - Histological diagnosis of IgAN - 24h-PER 1.0-3.5 g/day - No treatment with steroids or cytotoxic drugs during the previous 3 years |
|  |  |  | Supportive therapy (diuretics, antihypertensive and antiplatelet agents) (N=43) |  |
| Katafuchi et al.(67) | Prospective  Single-center  RCT  Open-label | Japan | Prednisolone [7.5-20 mg] (N=43) | - Aged <60 years - IgAN of glomerular score 4 to 7 - No previous treatment with steroids |
|  |  |  | Control (N=45) |  |
| Katafuchi et al.(68) |  |  | Prednisolone [7.5-20 mg] (N=43) |  |
|  |  |  | Control (N=47) |  |
| Ballardie and Roberts (69) | Prospective  Single-center  RCT  Controlled  Open-label | UK | Prednisolone [40 mg/day] + cyclophosphamide [1.5 mg/kg/day] + azathioprine [1.5 mg/kg/day] (N=19) | - Controlled hypertension - Isolated primary IgAN diagnosis - Aged <60 years - No previous immunosuppressive or corticosteroid treatment |
|  |  |  | Control, no immunosuppression (N=19) |  |
| Locatelli et al.(70) | Multicenter,  Open-label  RCT  controlled | Italy | MP [1 g] + prednisone [0.5 mg/kg] (N=43) | - Aged 15–69 years - Histological diagnosis of IgAN - 24h-PER 1.0–3.5 g/day - Plasma creatinine concentrations <133 μmol/L - No treatment with steroids or cytotoxic drugs during the previous 3 years |
|  |  |  | Supportive treatment (diuretics, antihypertensive drugs and antiplatelet agents) (N=43) |  |
| Mathur et al.(71); NCT04287985 | Phase 2  Multicenter  Double-blind  RCT | International | Sibeprenlimab [2 mg/kg] (N=38) | - 18 years of age or older - Biopsy-confirmed IgAN - PCR of at least 0.75 g/g - eGFR of at least 30 ml/min/1.73m² - Serum IgG level of at least 700 mg/dL - IgM level of at least 37 mg/dL - IgA level of at least 70 mg/dL - Receiving the highest stable dose of treatment with an ACEi or an ARB for at least 3 months before screening |
|  |  |  | Sibeprenlimab [4 mg/kg] (N=41) |  |
|  |  |  | Sibeprenlimab [8 mg/kg] (N=38) |  |
|  |  |  | Placebo (N=38) |  |
| Zhang et al.(72) | Prospective  RCT  Open-labelled | China | Leflunomide plus low-dose prednisone (n=59) | - Patients with biopsy-confirmed IgAN at a risk of progression |
|  |  |  | Prednisone alone (n=49) |  |
| Trial record for NCT03841448(73) | Phase 2  Randomized  Double-blind  Placebo-controlled | International | Total (N=31) | - Diagnosed with primary IgAN - Currently being treated for IgAN with stable, optimal therapy, including an ACEi or ARB - Has urine protein greater than or equal to 1 g/24h - Has hematuria (blood cells present in urine) |
|  |  |  | Cemdisiran (N=22) |  |
|  |  |  | Placebo (N=9) |  |
| Lai et al.(74) | RCT  Prospective  Open-label | Hong Kong | Prednisolone/ prednisone [40-60 mg/day] (N=17) | - Adult patients - Presence of predominant IgA deposits |
|  |  |  | No corticosteroid therapy (N=17) |  |
| Wheeler et al.(75); DAPA-CKD/ NCT03036150 | Phase 3  Double-blind  Placebo-controlled  RCT | International | Dapagliflozin [10 mg/day] (N=137) | - Adult patients - eGFR 25-75 mL/min/1.73 m² - UA/C 200-5000 mg/g - Receiving a stable dose of an ACEi or ARB - No immunotherapy for primary or secondary kidney disease within the previous 6 months before trial enrollment |
|  |  |  | Placebo (N=133) |  |
| Heerspink et al.(76); NCT03762850 | Phase 3  RCT  Double-blind  Parallel | International | Sparsentan [400mg/day] (N=202) | - Adult patients with biopsy-proven IgAN - 24-hour PER 1.0 g/day or higher at screening despite at least 12 weeks with stable dose of RAS inhibition - eGFR ≥30 mL/min/1.73 m² at screening |
| Rovin et al.(77); NCT03762850 |  |  | Irbesartan [300 mg/day] (N=202) |  |
| Lafayette et al.(78); ORIGIN/ NCT04716231 | Phase 2b  RCT  Double-blind  Placebo-controlled | NR | Atacicept [150 mg] (N=33) | - Patients with biopsy-proven IgAN - 24h urine protein >0.75 g/day or urine protein-to-creatinine ratio (UPCR) >0.75 g/g - eGFR >30 mL/min/1.73 m² despite optimized renin-angiotensin system blockade |
|  |  |  | Atacicept [75 mg] (N=33) |  |
| Barratt et al.(79); ORIGIN/ NCT04716231 |  |  | Atacicept [25 mg] (N=16) |  |
|  |  |  | Placebo (N=34) |  |
| Tam et al.(80); NCT02112838 | Phase 2  RCT  Placebo-controlled  Double-blind | International | Placebo (N=25) | - Adult patients - Biopsy-confirmed IgAN - Treatment with an ACEi and/or an ARB for at least 90 days - 24h-PER >1 g/day - eGFR >30 ml/min/1.73 m² - No recent use of cyclophosphamide, MMF, azathioprine, or rituximab; use of >15 mg/day prednisone (or other corticosteroid equivalent) |
| Tam et al.(81); NCT02112838 |  |  | Fostamatinib [100 mg] (N=26) |  |
|  |  |  | Fostamatinib [150 mg] (N=25) |  |
| Trial record for NCT02112838 (82) |  |  |  |  |
| Li et al.(83); ChiCTR1800014442 | Prospective  open-label  RCT | China | Methylprednisolone combined with alternative low-dose prednisone (MCALP; N=45) | - Diagnosed with IgAN by renal biopsy - Age of 15 to 75 years - Urinary protein excretion ≥1.0 g/24h and ≤3.5 g/24h - Stable blood pressure after comprehensive supportive care for at least 90 days - Serum creatinine <2.0 mg/dL (171 μmol/L) - eGFR-EPI >30 ml/min/1.73 m² |
|  |  |  | Full-dose prednisone (FP; N=42) |  |
| Ye et al.(84); NCT00426348 | Phase 4  Multicenter  Open-label  RCT  Controlled | China | Probucol [750 mg/day] + valsartan [160 mg/day] (N=34) | - Aged 18–75 years - 24h-PER 1.0–3.0 g/day - No treatment with an ACEI, ARB, anti-oxidant, lipid-lowering drug in the previous 6 weeks - No treatment with steroid or cytotoxic drug within the previous 6 months |
|  |  |  | Valsartan [160 mg/day] (N=35) |  |
| Shi et al.(85); NCT00793585 | Prospective  Open-label  RCT | China | Allopurinol [100-300 mg/day] (N=21) | - Aged 18–70 years - Biopsy-proven IgAN - 24h-PER 0.15-2.0 g/day - No prednisone or immunosuppressive drugs within 2 months prior to randomization - No ACEi and/or ARB |
|  |  |  | Control (N=19) |  |
| Kanjanabuch et al.(86) | RCT  Double-blind | Thailand | Pioglitazone [30 mg/day] (N=21) | - Biopsy-proven IgAN - Macroalbuminuria (24h-PER >0.5 g/day) - Had at least 2 risk factors for progressive disease - No immunosuppressive agents or steroid (>10 mg/day of prednisolone) |
|  |  |  | Placebo (N=20) |  |
| Chen et al.(87) | Prospective  RCT  Controlled  Open-label | China | Benazepril [10 mg/day] (N=36) | - Adult patients - Biopsy-proven IgAN - SCr <354 μmol/L - No prior ACEi or urokinase treatment |
|  |  |  | Benazepril [10 mg/day] + urokinase (N=35) |  |
| Sato et al.(88) | Preliminary  RCT  Controlled  Open-label | Japan | SCG [1,200 mg/day] (N=15) | - Adult patients - 24h-PER >1.0 g/day - IgAN diagnosis |
|  |  |  | SCG-responders |  |
|  |  |  | SCG-non-responders |  |
|  |  |  | Control (N=15) |  |
| Shima et al.(89); C000000363 | Prospective  Open-label  RCT | Japan | Prednisolone [2 mg/kg/day] + mizoribine [4 mg/kg/day] + warfarin [per day] + dipyridamole [6 mg/kg/day] (N=34) | - Aged 2 to 18 years - No previous treatment with corticosteroids or immunosuppressive drugs - Proteinuria >0.3 g/dL |
|  |  |  | Prednisolone [2 mg/kg/day] + mizoribine [4 mg/kg/day] (N=36) |  |
| Wu et al.(90); ChiCTR‑TRC‑10000776 | Prospective  Multicenter  Double‑dummy  RCT | China | Telmisartan (N=100) | - Aged 18–55 years - Biopsy-confirmed IgAN of Lee’s histological grade II–IV - 24h-PER 0.5-3.5 g/day - No use of corticosteroids or other immunosuppressive agents (including leflunomide) in the preceding 3 months |
|  |  |  | Telmisartan + clopidogrel (N=100) |  |
|  |  |  | Telmisartan + leflunomide (N=100) |  |
|  |  |  | Telmisartan + clopidogrel + leflunomide (N=99) |  |
| Cheng et al.(91) | Single-center  Double-blind  RCT | China | Valsartan [80 mg/day] (N=42) | - Aged between 18–55 years - Primary progressive IgAN diagnosed by pathology - Lee’s histological grade II–IV - 24h-PER 0.5-3.5 g/day - SCr <265.2 μmol/L - No use of cortisol or immunosuppressant agents within the last 3 months |
|  |  |  | Valsartan [80 mg/day] + clopidogrel [75 mg/day] (N=42) |  |
|  |  |  | Valsartan [80 mg/day] + leflunomide [20 mg/day] (N=42) |  |
|  |  |  | Valsartan [80 mg/day] + clopidogrel [75 mg/day] + leflunomide [20 mg/day] (N=42) |  |
| Xie et al.(92); CRG030600070 | Prospective  Multicenter  Open-label  RCT | China | Mizoribine [200-250 mg/day] + losartan [100 mg/day] (N=34) | - Aged 14-70 years - Biopsy-confirmed IgAN - 24h-PER 0.5-3.5 g/day - SCr <353.6 µmol/L - No use of steroids, immunosuppressants, ACEi and ARBs drug within the 3-month period preceding the study |
|  |  |  | Mizoribine [200-250 mg/day] (N=35) |  |
|  |  |  | Losartan [100 mg/day] (N=30) |  |
| Yoshikawa et al.(93) & Kamei et al.(94) | Prospective  Multicenter  RCT  Controlled  Double-blind | Japan | Prednisolone [2 mg/kg] + azathioprine [2 mg/kg/day] + heparin-warfarin + dipyridamole [5 mg/kg/day] (N=40) | - Aged 15 years - Newly diagnosed to have IgAN at study entry - No previous treatment with corticosteroids or immunosuppressive drugs |
|  |  |  | Heparin-warfarin + dipyridamole [5 mg/kg/day] (N=38) |  |
| Yoshikawa et al.(95) | Prospective  Multicenter  Open-label  RCT  Controlled | Japan | Prednisolone [2 mg/kg/day] + azathioprine [2 mg/kg/day] + warfarin + dipyridamole [5 mg/kg/day] (N=40) | - Aged 15 years - Newly diagnosed IgAN - No previous treatment with corticosteroids or immunosuppressive drugs |
|  |  |  | Prednisolone [2 mg/kg/day] (N=40) |  |
| Cheng et al.(96) | Prospective  Parallel  RCT  Open-label | Hong Kong | Captopril [12.5 mg/day] (N=12) | - Aged 21-65 years - Biopsy-proven IgAN - 24h-PER ≥ 1 g/day - Presence of tubulointerstitial scarring, tubular atrophy and global or segmental glomerulosclerosis |
|  |  |  | Captopril [12.5 mg/day] + ticlopidine [500 mg/day] (N=19) |  |
|  |  |  | Nadolol [40 mg/day] (N=16) |  |
| Woo et al.(97) & Woo et al.(98) | Double-arm  Prospective  RCT  Open-label | Singapore | Cyclophosphamide [1.5 mg/kg/day] + dipyridamole [300 mg/day] + warfarin (N=27) | - Adult patients - Diagnosis of mesangial IgAN |
|  |  |  | Control (N=21) |  |
|  |  |  | Continuation of dipyridamole [300 mg/day] + warfarin (N=13) |  |
|  |  |  | Control continuation (N=14) |  |
| Walker et al.(99) | RCT  Prospective  Controlled trial  Open-label | Australia | Cyclophosphamide [1-2 mg/kg/day] + dipyridamole [100 mg/day] + warfarin (N=25) | - Adult patients - Biopsy-proven IgAN - 24h-PER >1.0 g/day - SCr >0.12 mmol/L - Renal biopsy showing that >10% glomeruli contained crescents |
|  |  |  | Control (N=27) |  |

**Abbreviations**: 24h-PER, 24-hour urinary protein; ACEi, angiotensin-converting enzyme inhibitor; ARB, angiotensin receptor blocker; CCB, calcium channel blocker; CKD, chronic kidney disease; CSA, cyclosporine A; eGFR, estimated glomerular filtration rate; FU, follow-up; IgA, immunoglobulin A; IgAN, IgA nephropathy; IgG, immunoglobulin G; IgM, immunoglobulin; IQR, interquartile range; MTD, maximum tolerated dose; MMF, mycophenolate mofetil; MP, methylprednisolone; NR, not reported; PCR, protein creatinine ratio; RAS, renin-angiotensin; RASi, renin-angiotensin inhibitor; RCT, randomized controlled trial; SCr, serum creatinine; SD, standard deviation; SEM, standard error of the mean; UPCR, urine protein creatinine ratio.
