# Supplementary material for: Clinical study outcomes in IgA nephropathy: A systematic literature review and narrative synthesis
Source: PLoS One. 2025 Jun 10;20(6):e0323530. doi: 10.1371/journal.pone.0323530 (PMC12151485; doi:10.1371/journal.pone.0323530)
Supplement: S5 Table — (DOCX) [file pone.0323530.s005.docx]

**Supplementary Table S5:** Risk of bias assessment for all included RCTs

| **Author** | **Trial name/ NCT number** | **Random sequence generation** | **Allocation concealment** | **Similarity of groups at the outset of study** | **Blinding of participants and personnel** | **Drop-outs imbalances between groups** | **Measuring more outcomes than reported** | **Inclusion of ITT analysis/missing data** | **Declaration of conflict of interest** | **Total score** |
| --- | --- | --- | --- | --- | --- | --- | --- | --- | --- | --- |
| Mathur et al.(1) | NCT04287985 | 1 | 1 | 1 | 1 | 1 | 1 | 1 | 1 | 8 |
| Zhang et al.(2) | NCT03373461 | 1 | 1 | 1 | 1 | 1 | 1 | 0 | 1 | 7 |
| Barratt et al.(3) |  |  |  |  |  |  |  |  |  |  |
| - (4) |  |  |  |  |  |  |  |  |  |  |
| Barratt et al.(5) | NCT04564339 | 0 | 0 | 0 | 0 | 0 | 0 | 0 | -1 | -1 |
| Heerspink et al.(6) | NCT03762850 | 1 | 1 | 1 | 1 | 1 | 1 | 1 | 1 | 8 |
| Rovin et al.(7) |  |  |  |  |  |  |  |  |  |  |
| Hou et al.(8) | NCT01854814 | 1 | 0 | 1 | 1 | -1 | 1 | 1 | 1 | 5 |
| Kim et al.(9) | TESTING/ NCT01560052 | 1 | 1 | 1 | 1 | 1 | 1 | 1 | -1 | 6 |
| Kim et al.(10) |  |  |  |  |  |  |  |  |  |  |
| Lv et al.(11) |  |  |  |  |  |  |  |  |  |  |
| Lv et al.(12) |  |  |  |  |  |  |  |  |  |  |
| Lafayette et al.(13) | NCT03643965/ NefIgArd | 1 | 1 | 1 | 1 | -1 | 1 | 1 | 1 | 6 |
| Barratt et al. (2022) (14) |  |  |  |  |  |  |  |  |  |  |
| Barratt et al.(15) |  |  |  |  |  |  |  |  |  |  |
| Lafayette et al.(16) | NCT04716231 | 0 | 0 | 0 | 1 | 0 | 1 | 1 | 1 | 4 |
| Barratt et al.(17) |  |  |  |  |  |  |  |  |  |  |
| Lv et al.(18) | NCT04291781 | 1 | 0 | 1 | 1 | -1 | 1 | 1 | 1 | 5 |
| Lv et al.(19) |  |  |  |  |  |  |  |  |  |  |
| Sun et al.(20) | NR | 0 | 0 | 1 | 1 | 1 | 1 | 1 | 1 | 6 |
| Tam et al.(21) | NCT02112838 | 1 | 1 | 1 | 1 | 1 | 1 | 1 | 1 | 8 |
| Tam et al.(22) |  |  |  |  |  |  |  |  |  |  |
| - (23) |  |  |  |  |  |  |  |  |  |  |
| - (24) | NCT03841448 | 1 | 1 | 1 | 1 | 1 | 0 | 1 | -1 | 5 |
| Han et al.(25) | - | 1 | 0 | 1 | 0 | 1 | 1 | 0 | 1 | 5 |
| Jung et al.(26) |  |  |  |  |  |  |  |  |  |  |
| Li et al.(27) | ChiCTR1800014442 | 1 | 0 | 1 | -1 | 1 | 1 | 0 | 1 | 4 |
| Liang et al.(28) | NCT02160132 | 1 | 0 | 1 | -1 | -1 | 1 | 0 | 1 | 2 |
| Zhang et al.(29) | NR | 0 | 0 | 1 | 0 | 0 | 1 | 0 | -1 | 1 |
| Ni et al.(30) | ISRCTN97636235 | 1 | 1 | 1 | 1 | -1 | 1 | 1 | 1 | 6 |
| Wheeler et al.(31) | DAPA-CKD/ NCT03036150 | 1 | 0 | 1 | 1 | 1 | 1 | 1 | -1 | 5 |
| Lennartz et al.(32) | STOP-IgAN | 1 | 1 | 1 | -1 | 1 | 1 | 1 | -1 | -1 |
| Lennartz et al.(33) |  |  |  |  |  |  |  |  | 0 | 0 |
| Rauen et al.(34) |  |  |  |  |  |  |  |  | -1 | 4 |
| Rauen et al.(35) |  |  |  |  |  |  |  |  |  |  |
| Liu et al.(36) | NCT02942381 | 1 | 1 | 1 | 1 | -1 | 1 | 1 | 1 | 6 |
| Shima et al.(37) | C000000006 | 1 | 0 | 1 | 0 | 1 | 1 | 1 | -1 | 4 |
| Kohagura et al.(38) | ACTRN12610000516088 | 1 | 0 | 1 | -1 | 1 | 1 | 1 | 1 | 5 |
| Shima et al.(39) | C000000363 | 1 | 0 | 1 | 0 | -1 | 1 | 1 | -1 | 2 |
| Fellstrom et al.(40) | NEFIGAN/ NCT01738035 | 1 | 1 | 1 | 1 | 1 | 1 | 1 | -1 | 6 |
| Hirai et al.(41) | - | 1 | -1 | 1 | -1 | 1 | 1 | 1 | 1 | 4 |
| Hou et al.(42) | NCT01269021 | 1 | 1 | 1 | 1 | 1 | 1 | 1 | 1 | 8 |
| Lafayette et al.(43) | NCT00498368 | 1 | 0 | 1 | -1 | -1 | 0 | 1 | 1 | 2 |
| Min et al.(44) | - | 1 | 0 | 1 | 0 | -1 | 1 | 1 | 1 | 4 |
| Yu et al.(45) | NCT01224028 | 1 | 1 | 1 | 1 | 1 | 1 | 1 | 1 | 1 |
| Kim et al.(46) |  |  |  |  |  |  |  |  | 0 | 7 |
| Jo et al.(47) | - | 1 | 1 | 1 | 0 | -1 | 1 | 1 | 1 | 5 |
| Masutani et al.(48) | UMIN000000593 | 1 | -1 | 1 | 0 | 1 | 1 | 1 | -1 | 3 |
| Wu et al.(49) | ChiCTR‑TRC‑10000776 | 1 | 1 | 1 | 1 | -1 | 1 | 1 | 1 | 6 |
| Cheng et al.(50) | - | 1 | 1 | 1 | 1 | 1 | 1 | 1 | 1 | 8 |
| Hogg et al.(51) | NCT00318474 | 0 | -1 | 1 | -1 | -1 | 1 | 1 | 1 | 1 |
| Liu et al.(52) | - | 1 | 0 | 1 | 0 | 1 | 1 | 1 | 1 | 6 |
| Liu et al.(53) | - | 0 | 0 | 1 | 0 | 1 | 1 | 1 | 1 | 5 |
| Ye et al.(54) | NCT00426348 | 1 | 0 | 1 | 1 | 1 | 1 | 1 | 0 | 6 |
| Li et al.(55) | NCT01225445 | 1 | 1 | 1 | -1 | 1 | 1 | 1 | 1 | 6 |
| Pozzi et al.(56) | - | 1 | 0 | 1 | -1 | 1 | 1 | 1 | 1 | 5 |
| Pozzi et al.(57) |  |  |  |  |  |  |  |  |  |  |
| Shi et al.(58) | NCT00793585 | 1 | 1 | 1 | -1 | 1 | 1 | 1 | -1 | 4 |
| Kamei et al.(59) | - | 1 | 0 | 1 | -1 | -1 | 1 | 1 | 1 | 1 |
| Yoshikawa et al.(60) |  |  |  |  |  |  |  |  | 0 | 2 |
| Xie et al.(61) | CRG030600070 | 0 | 0 | 1 | 0 | -1 | 1 | 1 | 0 | 2 |
| Liu et al.(62) | - | 0 | 0 | 1 | 0 | 1 | 1 | 1 | 0 | 4 |
| Tang et al.(63) | NCT00863252 | 1 | 0 | 1 | 0 | 1 | 1 | 1 | 1 | 1 |
| Tang et al.(64) |  |  |  |  |  |  |  |  | 0 | 5 |
| Kanjanabuch et al.(65) | - | 1 | 1 | 1 | 1 | 0 | 0 | 1 | 1 | 6 |
| Lv et al.(66) | NCT00378443 | 1 | 0 | -1 | 0 | 1 | 1 | 1 | 1 | 4 |
| Manno et al.(67) | - | 1 | -1 | 1 | -1 | 1 | 1 | 1 | 1 | 4 |
| Woo et al.(68) | - | 1 | 0 | 1 | 0 | -1 | 1 | 1 | 1 | 4 |
| Koike et al.(69) | - | 1 | 0 | 1 | -1 | 1 | 1 | 1 | 0 | 4 |
| Shimizu et al.(70) | - | 0 | 1 | 1 | 0 | 1 | 1 | 0 | 0 | 4 |
| Coppo et al.(71) | IgACE | 1 | 1 | 1 | 1 | 1 | 1 | 1 | 1 | 8 |
| Horita et al.(72) | - | 0 | 0 | 1 | -1 | 1 | 1 | 1 | 0 | 3 |
| Woo et al.(73) | - | 1 | 0 | 1 | 0 | 1 | 1 | 1 | 0 | 5 |
| Horita et al.(74) | - | 0 | 0 | 1 | -1 | 1 | 1 | 1 | 0 | 3 |
| Horita et al.(75) |  |  |  |  |  |  |  |  |  |  |
| Li et al.(76) | HKVIN study | 1 | 1 | 1 | 1 | -1 | 1 | 1 | 1 | 6 |
| Lou et al.(77) | - | 0 | 0 | 1 | 0 | 1 | 1 | 1 | 0 | 4 |
| Yoshikawa et al.(78) | - | 1 | 0 | 1 | -1 | -1 | 1 | 1 | 0 | 2 |
| Frisch et al.(79) | - | 1 | 1 | 1 | 1 | 1 | 1 | 1 | -1 | 6 |
| Kanno et al.(80) | - | 0 | 0 | 1 | 0 | 0 | 0 | -1 | 0 | 0 |
| Chen et al.(81) | - | 0 | 0 | 1 | 0 | 1 | 1 | 1 | 1 | 5 |
| Maes et al.(82) | - | 1 | 0 | 1 | 0 | 1 | 1 | 1 | 0 | 5 |
| Pozzi et al.(83) | - | 1 | 0 | 1 | 0 | 1 | 1 | 1 | 0 | 5 |
| Pozzi et al.(84) |  |  |  |  |  |  |  |  |  |  |
| Katafuchi et al.(85) | - | 1 | -1 | 1 | -1 | 1 | 1 | 1 | 0 | 3 |
| Katafuchi et al.(86) |  |  |  |  |  |  |  |  |  |  |
| Kim et al.(87) | - | 0 | 0 | 1 | 1 | 1 | 1 | 1 | 0 | 5 |
| Park et al.(88) | - | 0 | 0 | 1 | 0 | 1 | 1 | 1 | 1 | 5 |
| Praga et al.(89) | - | 1 | 1 | 1 | 0 | 1 | 1 | 1 | 0 | 6 |
| Ballardie et al.(90) | - | 0 | 0 | 1 | 0 | 1 | 1 | 1 | 0 | 4 |
| Locatelli et al.(91) | - | 0 | 0 | 1 | 0 | -1 | 1 | 1 | 0 | 2 |
| Nakamura et al.(92) | - | 0 | 0 | 1 | 1 | 1 | 1 | 1 | 0 | 5 |
| Woo et al.(93) | - | 0 | 0 | 1 | 0 | 1 | 1 | 1 | 0 | 4 |
| Cheng et al.(94) | - | 1 | 0 | 1 | 0 | 1 | 1 | 1 | -1 | 4 |
| Maschio et al.(95) | - | 0 | 0 | 0 | 1 | 1 | 1 | 1 | 0 | 4 |
| Woo et al.(96) | - | 0 | 0 | 1 | -1 | 0 | 1 | 1 | 0 | 2 |
| Woo et al.(97) |  |  |  |  |  |  |  |  |  |  |
| Sato et al.(98) | - | 0 | 0 | 1 | 0 | 1 | 1 | 1 | 0 | 4 |
| Walker et al.(99) | - | 1 | 0 | 1 | 0 | 1 | 1 | 1 | 0 | 5 |
| Lai et al.(100) | - | 0 | 0 | 1 | 0 | 1 | 1 | 1 | 0 | 4 |

**Abbreviations**: ITT, intention to treat; NCT, National Clinical Trial; RCT, randomized controlled trial.

**Summary of answers to each question:**

**Was the method used to generate random allocations adequate?** Yes (green) = low risk of bias. No (red) = high risk of bias. Not applicable or not clear (yellow) = unclear risk of bias

**Was the allocation adequately concealed?** Yes (green) = low risk of bias. No (red) = high risk of bias. Not applicable or not clear (yellow) = unclear risk of bias

**Were the groups similar at the outset of the study in terms of prognostic factors, for example, severity of disease?** Yes (green) = low risk of bias. No (red) = high risk of bias. Not applicable or not clear (yellow) = unclear risk of bias

**Were the care providers, participants, and outcome assessors blind to treatment allocation? If any of these people were not blinded, what might be the likely impact on the risk of bias (for each outcome)?** Yes (green) = low risk of bias. No (red) = high risk of bias. Not applicable or not clear (yellow) = unclear risk of bias

**Were there any unexpected imbalances in drop-outs between groups? If so, were they explained or adjusted for?** No (green) = low risk of bias. Yes (red) = high risk of bias. Not applicable or not clear (yellow) = unclear risk of bias

**Is there any evidence to suggest that the authors measured more outcomes than they reported?** No (green) = low risk of bias. Yes (red) = high risk of bias. Not applicable or not clear (yellow) = unclear risk of bias

**Did the analysis include an intention to treat analysis? If so, was this appropriate and were appropriate methods used to account for missing data?** Yes (green) = low risk of bias. No (red) = high risk of bias. Not applicable or not clear (yellow) = unclear risk of bias

**Did the study authors declare any conflicts of interest? (Yes indicates the inclusion of a declaration of interest regardless of the presence or absence of a conflict)** Yes (green) = low risk of bias. No (red) = high risk of bias. Not applicable or not clear (yellow) = unclear risk of bias
