# Supplementary material for: Clinical study outcomes in IgA nephropathy: A systematic literature review and narrative synthesis
Source: PLoS One. 2025 Jun 10;20(6):e0323530. doi: 10.1371/journal.pone.0323530 (PMC12151485; doi:10.1371/journal.pone.0323530)
Supplement: S6 Table — (DOCX) [file pone.0323530.s006.docx]

**Supplementary Table S6:** Risk of bias assessment for all included non-randomized studies

| **Author;**  **Trial identifier** | **Selection** | | | | **Comparability** | **Outcome** | | | **Total stars** |  |
| --- | --- | --- | --- | --- | --- | --- | --- | --- | --- | --- |
|  | **Q1** | **Q2** | **Q3** | **Q4** | **Q1** | **Q1** | **Q2** | **Q3** |  |  |
| Andreoli, et al. (1) | a* | NA | a* | a* | a* | d | a* | a* | 6 |  |
| Bazzi, et al. (2) | b | a* | d | a* | a* | d | a* | a* | 5 |  |
| Bomback, et al. (3) NCT01129284 | d | a* | a* | a* | NA | b* | b | a* | 5 |  |
| Bruchfeld et al (4)  NCT02384317 | b* | NA | a* | a* | NA | d | a* | a* | 5 |  |
| Choi, et al. (5) | a* | NA | a* | a* | NA | d | a* | a* | 5 |  |
| Clarkson, et al. (6) | a* | a* | d | a* | a* | d | a* | a* | 6 |  |
| Eigido, et al. (7) | d | b | d | a* | a* | d | a* | d | 3 |  |
| Fang, et al. (8) | a* | NA | a* | a* | NA | d | a* | a* | 5 |  |
| Grcevska, et al. (9) | d | c | a* | b | b* | b* | a* | d) | 4 |  |
| Hartono, et al. (10) NCT01103778 | b* | NA | d | a* | NA | d | a* | b* | 4 |  |
| Hotta, et al. (11) | d | c | d | b | NA | d | a* | a* | 2 |  |
| Ikeda, et al. (12) | b* | NA | d | a* | NA | d | a* | a* | 4 |  |
| Kaneko, et al. (13) | d | b | d | a* | a* | d | b | a* | 3 |  |
| Kawasaki, et al. (14) | b* | NA | a* | a* | a* | d | a* | a* | 6 |  |
| Kim, et al. (15) | d | NA | a* | a* | NA | b* | a* | a* | 5 |  |
| Kim, et al. (16) | a* | a* | a* | a* | a* | b* | a* | a* | 8 |  |
| Kim, et al. (17)  Rastogi, et al. (18)  AFFINITY | a* | NA | a* | a* | NA | d | a* | a* | 5 |  |
| Kobayashi, et al. (19) | a* | a* | a* | a* | a* | b* | a* | a* | 8 |  |
| Komatsu, et al. (20) | a* | a* | a* | a* | a* | d | a* | a* | 7 |  |
| Koyama, et al. (21) | a* | a* | a* | a* | a* | b* | a* | a* | 8 |  |
| Lafayette, et al. (22) NCT02682407 | b* | NA | a* | a* | NA | d | a* | a* | 5 |  |
| Lingaraj, et al. (23) | b* | NA | d | b | NA | d | b | d | 1 |  |
| Lundberg, et al. (24) | b* | NA | a* | a* | NA | d | a* | a* | 5 |  |
| Nagaoka, et al. (25) | a* | NA | d | a* | NA | d | a* | a* | 4 |  |
| Nakamura, et al. (26) | c | a* | d | a* | a* | d | a* | a* | 5 |  |
| Nakanishi, et al. (27) | a* | NA | d | a* | NA | d | a* | b* | 4 |  |
| Palla, et al. (28) | c | NA | b* | a* | a* | b* | a* | a* | 6 |  |
| Pergola, et al. (29) PHOENIX  NCT03366337 | b* | NA | a* | a* | NA | b* | a* | a* | 6 |  |
| Rasche, et al. (30) Rasche, et al. (31) Rasche, et al. (32) Rasche, et al. (33) | a* | NA | b* | a* | a* | b* | a* | a* | 7 |  |
| Rekola, et al. (34) | b* | a* | a* | a* | a* | b* | a* | a* | 8 |  |
| Remuzzi, et al. (35) | d | d | d | b | NA | d | a* | d | 1 |  |
| Roccatello, et al. (36) | d | b | d | a* | Not clear | d | a* | d | 2 |  |
| Roccatello, et al. (37) | b* | a* | a* | a* | a* | b* | b | a* | 7 |  |
| Roccatello, et al. (38) | b* | NA | d | a* | NA | d | a* | a* | 4 |  |
| Rostoker, et al. (39) | a* | NA | a* | a* | a* | b* | a* | a* | 7 |  |
| Russo, et al. (40) | a* | NA | a* | a* | a* | b* | a* | a* | 7 |  |
| Segarra, et al. (41) | a* | NA | a* | a* | NA | d | a* | d | 4 |  |
| Sugiura, et al. (42) | d | NA | a* | a* | NA | d | a* | a* | 4 |  |
| Tanaka, et al. (43) | b* | a* | d) | a* | a* | d | a* | a* | 6 |  |
| Tang, et al. (44)  NCT00922311 | b* | NA | d | a* | NA | d | a* | b* | 4 |  |
| Tasanarong, et al. (45) | b* | NA | a* | a* | NA | d | a* | a* | 5 |  |
| Tomino, et al. (46) | b* | a* | d | a* | a* | d | a* | a* | 6 |  |
| Tomino, et al. (47) | a* | NA | d | a* | NA | d | a* | a* | 4 |  |
| Trimarchi, et al. (48) | a* | NA | d | a* | NA | d | a* | a* | 4 |  |
| Tumlin, et al. (49) | b* | a* | d | a* | a* | b* | a* | a* | 7 |  |
| Uzu, et al. (50) | a* | NA | d | a* | a* | d | a* | a* | 5 |  |
| Waldo, et al. (51) | b* | b | d | b | NR | d | a* | d | 2 |  |
| Wang, et al. (52) NCT01451710 | b* | NA | d | a* | NA | d | a* | d | 3 |  |
| Xu, et al. (53) | a* | a* | d | a* | a* | d | b | b* | 5 |  |
| Xydakis, et al. (54) | b* | NA | d | a* | NA | d | a* | a* | 4 |  |
| Yagi, et al. (55) | a* | a* | d | a* | a* | d | a* | a* | 6 |  |
| Yoshikawa, et al. (56) | b* | NA | d | a* | NA | d | a* | b* | 4 |  |
| Yu, et al. (57) | a* | a* | a* | a* | a* | d | a* | a* | 7 |  |
| Zand, et al. (58) NCT02282930 | b* | NA | a* | a* | NA | d | b | a* | 4 |  |

Summary of risk of bias assessment questions

Selection

1. **Representativeness of the exposed cohort**
   1. Truly representative of the average (describe) in the community ٭
   2. Somewhat representative of the average in the community ٭
   3. Selected group of users e.g. Nurses, volunteers
   4. No description of the derivation of the cohort
2. **Selection of the non-exposed cohort**
   1. Drawn from the same community as the exposed cohort٭
   2. Drawn from a different source
   3. No description of the derivation of the non-exposed cohort
3. **Ascertainment of exposure**
   1. Secure record (e.g. Surgical records) ٭
   2. Structured interview ٭
   3. Written self-report
   4. No description
4. **Demonstration that outcome of interest was not present at start of study**
   1. Yes ٭
   2. No

**Comparability**

1. **Comparability of cohorts on the basis of the design or analysis**
   1. Study controls for *(select the most important factor)* ٭
   2. Study controls for any additional factor ٭ *(This criteria could be modified to indicate specific control for a second important factor.)*

**Outcome**

1. **Assessment of outcome**
   1. Independent blind assessment ٭
   2. Record linkage ٭
   3. Self-report
   4. No description
2. **Was follow-up long enough for outcomes to occur**
   1. Yes *(select an adequate follow up period for outcome of interest)* ٭
   2. No
3. **Adequacy of follow-up of cohorts**
   1. Complete follow up - all subjects accounted for ٭
   2. Subjects lost to follow up unlikely to introduce bias - small number lost - > ____ % *(select an adequate %) follow up, or description provided of those lost)* ٭
   3. Follow up rate < ____% *(select an adequate %)*
